# Misconduct in Research and Publication

**DOI:** 10.18869/acadpub.ibj.21.5.284

**Published:** 2017-09

**Authors:** Viroj Wiwanitkit

**Affiliations:** 1 Hainan Medical University, China; 2 Dr. DY Patil University, China

Dear Editor, I read the recent publication on “Misconduct in Research and Publication” with great interest[1]. I agree that misconduct in research and publication is not uncommon. Nevertheless, it is rarely mentioned. In fact, there are many incorrect conceptions among researchers on publication ethics. The milder examples are attempts to report only the “positive outcomes”, textual or data recycling as well as figure cropping and modification. These problems can be seen in published work of many academic members regardless of nationality or seniority. To provide the needed education and instructions aiming at primary prevention of the problem is widely practiced. A secondary means of prevention is screening for misconduct for the purpose of early detection. Nevertheless, such misconduct is repeatedly observed.

An important question is whether such measures are suitable corrective actions. In general, if the misconduct is detected, reporting to the researchers’ organization, as well as retraction of the articles with public announcement by the journal, are recommended. Nevertheless, there is often no incurred penalty or response from the researchers under question or their affiliated organizations. If those committing the misconducts are senior academics, they might receive no penalty and may even be further promoted based those questionable publications[2]. Sometimes, the journals, which are usually of poor quality and predatory type also support the misconduct incidence[2]. Such behavior sets fallacious examples for the rest. Of interest, although there are extensive attempts to promote anti-misconduct communities, which normally fail. A good example is the launching of Déjà vu database to combat plagiarism[3], which is currently non-functional. How to promote the ethical practice among the practitioners and promote the anti-misconduct community is a major challenge facing our scientific community.
